# NEIGHBORHOOD PERCEPTIONS AND SUICIDAL RISK BEHAVIORS: A CROSS-SECTIONAL STUDY AMONG OLDER CHINESE IMMIGRANTS

**DOI:** 10.1093/geroni/igae098.1376

**Published:** 2024-12-31

**Authors:** Chuwen Zhong, Weidi Qin, Yanping Jiang, Fengyan Tang, Viktoryia Kalesnikava

**Affiliations:** University of Michigan, Ann Arbor, Michigan, United States; University of Wisconsin Madison, Madison, Wisconsin, United States; Rutgers, The State University of New Jersey, New Brunswick, New Jersey, United States; University of Pittsburgh, Wexford, Pennsylvania, United States; University of Michigan, Ann Arbor, Michigan, United States

## Abstract

Compared to other age groups, older adults have a higher suicide risk. Studies have shown that the neighborhood environment contributes to psychosocial well-being. As the largest Asian subgroup in the U.S., Chinese immigrants make up 24% of the total Asian American population. However, it remains understudied how perceived neighborhood characteristics impact suicidal ideation and suicidal risk behaviors among older adults in this population. The study aims to investigate the association between perceived neighborhood characteristics and suicidal ideation and suicidal risk behaviors (i.e., hopelessness, depressive symptoms, and loneliness) among older Chinese immigrants. Data were drawn from the Population Study of Chinese Elderly (PINE), which was collected between 2011-2013. Neighborhood cohesion and disorder scales were used, Cronbach’s alpha= 0.86 and 0.80 respectively. The suicidal ideation was measured by lifetime suicidal ideation. Hopelessness was assessed by the 7-item Beck hopelessness scale, depressive symptoms were measured by the Patient Health Questionnaire (PHQ-9), and loneliness was measured by the R-UCLA loneliness scale. Linear and logistics regressions were conducted to test the study aims. Out of a total of 3,157 respondents, 58% were female with an average age of 72.8. Higher levels of neighborhood cohesion were associated with less suicidal ideation, loneliness, and depression, but not hopelessness. On the contrary, neighborhood disorder was positively associated with all outcome variables including hopelessness. This study suggests that the neighborhood environment plays a crucial role in older Chinese immigrants’ health and well-being. Culturally relevant and community-level efforts are needed to promote successful aging in place among this group.

